# High-Specific-Surface-Area Hollow Carbon Spheres for Efficient Chromium Ion Adsorption in Acidic Wastewater

**DOI:** 10.3390/nano16110669

**Published:** 2026-05-26

**Authors:** Rui Gao, Man Zhang, Xiaoyu Sun, Dongyang Zhu, Xin Huang, Ting Wang, Chuang Xie, Na Wang, Hongxun Hao

**Affiliations:** 1 National Engineering Research Center of Industry Crystallization Technology, School of Chemical Engineering and Technology, Tianjin University, Tianjin 300072, China; 2023207457@tju.edu.cn (R.G.); 2023207381@tju.edu.cn (M.Z.); 2023207090@tju.edu.cn (X.S.); zhudongyang@tju.edu.cn (D.Z.); x_huang@tju.edu.cn (X.H.); wang_ting@tju.edu.cn (T.W.); acxie@tju.edu.cn (C.X.); 2 State Key Laboratory of Chemical Engineering and Low-Carbon Technology, Shanghai 200237, China; 3 Engineering Research Center of Green Purification Process, Ministry of Education, Tianjin University, Tianjin 300072, China

**Keywords:** hollow carbon microspheres (HCM_2.5_), ultrahigh surface area, specific adsorption, metal ions

## Abstract

Carbon materials are regarded as cost-effective adsorbents due to their ability to remove heavy metals and organic pollutants from contaminated water. In this study, a novel phenol–formaldehyde resin-derived carbon microsphere (HCM_2.5_) was designed and synthesized via a hard-template method combined with KOH activation. The prepared HCM_2.5_ exhibits high selectivity and removal efficiency toward heavy metal ions and delivers an ultrahigh specific surface area of 2165 m^2^/g. A Cr(VI) removal efficiency exceeding 99.6% could be achieved in 50 ppm acidic solution, with excellent performance at pH 2–5. X-ray diffraction (XRD), Brunauer–Emmett–Teller (BET) nitrogen adsorption–desorption analysis, and scanning electron microscopy (SEM) were used to confirm its porous structure with a high specific surface area. The results of X-ray photoelectron spectroscopy (XPS) and Fourier transform infrared spectroscopy (FT-IR) reveal that the efficient heavy metal removal performance of HCM_2.5_ is mainly attributed to its high specific surface area, as well as coordination and redox reactions between oxygen-containing functional groups and heavy metal ions. Furthermore, benefiting from its outstanding specific surface area and well-developed pore structure, a physical–chemical synergistic adsorption mechanism was proposed and systematically elucidated.

## 1. Introduction

Nowadays, acidic wastewater (pH = 1–6) mainly originates from metal processing and surface treatment, chemical production (fertilizer manufacturing, organic synthesis, etc.), metal mining, battery manufacturing, the electronics industry (semiconductor cleaning [1]), coal coking [2], and other industries. Such wastewater is highly corrosive and typically contains metals such as Fe, Cu, Zn, Pb, and Cr, etc. Direct discharge of it would damage aquatic ecosystems, corrode pipelines, and degrade soil. Among various heavy metal species, hexavalent chromium (Cr(VI)) is extremely toxic and poorly biodegradable. Therefore, it is urgent to develop efficient methods for removing Cr(VI) from acidic wastewater.

In recent years, various techniques have been developed for the effective removal of heavy metals from wastewater, including ion exchange [3], crystallization [1], membrane separation [4], adsorption [5], solvent extraction [6], and photocatalysis [7], etc. Compared with other methods, adsorption has emerged as a particularly advantageous approach for removing Cr(VI) from acidic wastewater due to its high efficiency, simple operation, low cost, good selectivity, environmental friendliness, and wide applicability [8]. Moreover, a major advantage of this method is its potential to minimize sludge production and reduce the consumption of other reagents, thereby enhancing sustainability. Numerous studies have verified the feasibility of adsorption for Cr(VI) removal. For example, functionalized materials such as caffeic-acid-modified corn starch [9], chitosan/g-C_3_N_4_/TiO_2_ [10], and polypyrrole-coated molybdenum disulfide composites [11], have shown promise in binding Cr(IV) ions in solution. Understanding the adsorption mechanism of Cr(VI) under acidic conditions is crucial for improving overall removal performance [12].

Carbon materials have emerged as a focal point for removing heavy metals from acidic wastewater, mainly due to their exceptional resistance to strong acidic environments. Among various acid-resistant materials (chitosan [8,13], graphene oxide/reduced graphene oxide [14], and polymers [15], etc.), carbon materials [16,17] stand out for their sp^2^-hybridized carbon skeleton, which remains stable even in highly concentrated solutions of strong acids (HCl, H_2_SO_4_, HNO_3_). This resilience prevents dissolution, decomposition, or structural collapse during treatment of strongly acidic Cr-containing wastewater, making carbon materials highly effective adsorbents for Cr(VI). However, unactivated and unmodified carbon materials suffer from low specific surface area, unideal pore structure, and insufficient surface active sites. Consequently, their adsorption capacity and catalytic activity are limited, hindering the efficient removal of mixed pollutants in complex water matrices. Therefore, activation and surface modification of carbon materials are essential to boost performance and broaden their environmental remediation applications. Such enhancements will improve heavy metal removal efficiency and support more sustainable water-management solutions.

To overcome these drawbacks, various modification strategies have been developed, including physicochemical activation [18], heteroatom doping [19,20], structural and morphological regulation [21,22], and composite modification [23]. Among them, physicochemical activation and morphological control are recognized as the most effective approaches to enhance adsorption performance [24]. Activation by CO_2_ [18], steam, or chemical agents such as KOH [25], NaOH [26], ZnCl_2_ [27], and H_3_PO_4_ [28] could create highly developed hierarchical pores (micro-, meso-, and macropores) and large specific surface areas, providing abundant adsorption sites for pollutants. Fabricating carbon materials into nanospheres could effectively shorten mass transfer pathways, expose more active sites, and accelerate reaction rates. Well-formed carbon microspheres/particles also exhibit high mechanical strength, and could resist pulverization under pressure in continuous-flow adsorption columns, and could be easily separated via filtration or centrifugation, reducing long-term application costs. Recent studies show that alkali-activated carbon materials can significantly improve adsorption performance for heavy metal. For example, Zhao et al. [29] demonstrated that the particle size, shell thickness and pore size of carbon spheres can be precisely tuned based on reaction kinetics. The resulting high-carbon spheres (HCSs) exhibit high adsorption capacities for various contaminants, including VB_12_, MB and Cr(VI). Furthermore, Liang et al. [30] prepared a nitrogen-doped porous carbon material (PCM-N) via a two-step hydrothermal-activation method using KOH as the activating agent, which achieved an ultrahigh adsorption capacity for Cr(VI), underscoring the potential of these modified carbon materials for addressing heavy metal pollution in environmental applications. The synthesis of porous carbon spheres with high specific surface area is an effective way to improve adsorption capacity. The distinctive porous spherical architecture could reduce mass transfer distance, expose more active sites, and enhance reaction rates. Therefore, it is highly desirable to explore a facile method for preparing porous carbon spheres with high adsorption activity. Carbon spheres are generally synthesized by hydrothermal and template-based methods. Although hydrothermally prepared carbon spheres possess abundant functional groups and favorable physical properties [31], their tendency to aggregate can decrease the specific surface area and limit their applicability. Template-based approaches, by contrast, have emerged as effective routes for fabricating porous carbon spheres with high adsorption activity, enabling precise control over morphology and structure integrity. In particular, porous hollow carbon spheres prepared by hard-template method feature tunable pore structure, uniform morphology, large specific surface area, and excellent stability.

In this study, a novel phenol–formaldehyde resin-derived carbon microsphere (HCM_2.5_) was designed and synthesized via a hard-template method using monodisperse SiO_2_ microspheres as the template and resorcinol–formaldehyde as a carbon precursor, in combination with KOH activation. Moreover, the structure, morphology, adsorption performance, and stability of the activated porous hollow carbon microspheres were comprehensively characterized by Brunauer–Emmett–Teller (BET) N_2_ adsorption–desorption analysis, X-ray photoelectron spectroscopy (XPS), and Fourier transform infrared spectroscopy (FT-IR), etc. Results demonstrated that HCM_2.5_ could exhibit excellent adsorption performance toward Cr(VI) under acidic conditions. Finally, a detailed adsorption mechanism of Cr(VI) in acidic solution onto porous hollow carbon microspheres was proposed, enriching understanding of contaminant–adsorbent interactions.

## 2. Materials and Methods

### 2.1. Materials Preparation

**Figure 1:**
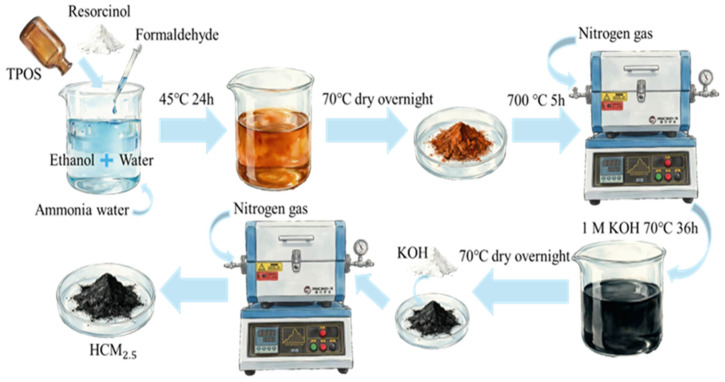
The synthesis process of HCM_2.5._

The synthesis process outlined in Figure 1 is a sequence of carefully controlled steps to prepare HCM and subsequently HCM_2.5_ and HCM_5_. It begins with the addition of 10.38 mL of tetrapropyl orthosilicate (TPOS) to a mixture of 180 mL ethanol, 9 mL ammonia solution, and 60 mL deionized water, with stirring at room temperature. Then, 1.2 g resorcinol and 1.68 mL formaldehyde are added, and the mixture is stirred for 24 h to allow thorough interaction of the reactants. After standing and filtration, the product is dried at 70 °C overnight to obtain SiO_2_@RF composite particles. The composite is calcined at 700 °C for 5 h under N_2_ flow, followed by etching with 1 M KOH solution at 70 °C to remove SiO_2_, and dried at 70 °C overnight to obtain HCM [29,32].

In the next phase, 0.6 g HCM is mixed with KOH at a KOH/HCM mass ratio of 2.5/5.0. The mixture was calcined at 650 °C for 1.25 h under N_2_ flow. The obtained solid is immersed in HCl solution for 30 min until no bubbles evolved, then dried at 70 °C overnight to yield HCM_2.5_ and HCM_5_.

### 2.2. Materials Characterization

X-ray diffraction (XRD) analysis: The crystal structure of the material was characterized using a Rigaku D/max 2500 X-ray diffractometer (Rigaku Corporation, Tokyo, Japan), equipped with a Cu target (λ = 0.15418 nm) under operating conditions of 40 kV and 100 mA. The diffraction patterns were recorded over a 2*θ* range of 2°–80° at a scanning rate of 8°/min.

Electron microscopy analysis: Electron microscopy analysis was conducted to investigate the morphology and structure of HCM*_x_*. Scanning electron microscopy (SEM) was performed using a Thermo Fisher Apreo S LoVac microscope (Thermo Fisher Scientific, Waltham, MA, USA), with samples sputter-coated with gold and an accelerating voltage of 10 kV. Transmission electron microscopy (TEM) was conducted using a Thermo Fisher Tecnai G2 F20 microscope (Thermo Fisher Scientific, Waltham, MA, USA) at an accelerating voltage of 200 kV.

X-ray photoelectron spectroscopy (XPS) analysis: Elemental composition and chemical states of HCM*_x_* were analyzed using a Thermo Fisher K-Alpha spectrometer (Thermo Fisher Scientific, Waltham, MA, USA), which utilized Al Kα radiation (1486.6 eV) with a beam spot size of 400 μm. The spectrometer was operated at a voltage of 12 kV, and a current of 6 mA. All binding energies were calibrated against the C 1s peak at 248.8 eV.

Surface area and pore structure analysis: A Kubo 1200 physisorption analyzer (Beijing Builder Electronic Technology Co., Ltd., Beijing, China) was employed to evaluate porosity. The Brunauer–Emmett–Teller (BET) method was used to calculate specific surface area, while the Barrett–Joyner–Halenda (BJH) model were used to determine the pore volume and average pore size.

Fourier transform infrared spectroscopy (FT-IR) analysis: Surface functional groups were characterized using a Bruker Vertex 70 spectrometer (Bruker Optics GmbH, Ettlingen, Germany) with a scanning range of 4000–400 cm^−1^.

Inductively coupled plasma optical emission spectroscopy (ICP-OES) analysis: The elemental concentration and impurity content of the material were quantified using an Agilent 7800 ICP-OES spectrometer (Agilent Technologies Inc., Santa Clara, CA, USA). The spectrometer was operated with a pump rate of 60 r/min, a nebulizer flow of 0.70 L/min, an auxiliary gas flow of 1.0 L/min, and a plasma gas flow of 12.0 L/min. The RF power was set at 1250 W, with a sample flush time of 20 s, a stable time of 20 s, and a reading access time of 5 s.

### 2.3. Adsorption Experiments of Cr(VI)

The adsorption performance of carbon-based materials, including HCM, HCM_5_ and HCM_2.5_, was systematically evaluated via a batch adsorption method. Solid sodium chromate (Na_2_CrO_4_) was used as the chromium source to prepare chromium-containing solutions. This process involved preparing a 20 mL glass vial containing a Cr(VI) solution mixed with a predetermined quantity of either HCM or HCM_2.5_ or HCM_5_. The pH of the solution was adjusted using hydrochloric acid or sodium hydroxide (0.01 M). Following the adsorption period, the solution was subjected to filtration through a 220 nm membrane filter, and the residual Cr(VI) concentration was quantified by ICP-OES [33]. The investigation considered critical experimental variables, including adsorption time (300–3600 min), initial Cr(VI) concentration (100–500 mg/L), pH (1–7), and temperature (25–45 °C). The method followed was the same as described previously and will not be detailed further here. A thermostatic shaker was used to maintain the desired temperature throughout the adsorption process. The adsorption capacity was calculated as follows [34]:

(1) $$q_{e}=\frac{(C_{2}-C_{1})V}{m}$$

where *q_e_* represents the equilibrium adsorption capacity (mg/g), *C_1_* is the initial concentration (mg/L), *C_2_* is the concentration of the adsorption solution (mg/L), *V* is the volume of the solution (L), and *m* is the mass of the adsorbent (g).

### 2.4. Reusability and Effectiveness of HCM_2.5_

In the recycling tests conducted, the Cr(VI) concentration was fixed at 100 mg/L, with an adsorbent dosage of 1 g/L and solution pH of 3. Following a 24 h adsorption period, the resulting solid was subjected to desorption in a 1M sodium hydroxide (NaOH) solution, also lasting 24 h. Subsequently, to ensure neutrality, the desorbed solid was treated with 1M HCl. After this neutralization step, the solid was filtered and subsequently dried in an oven at 70 °C for 12 h. The dried material underwent a thorough examination and was subsequently reused in the adsorption process described above, thereby allowing for an assessment of the material’s reusability and effectiveness in ongoing Cr(VI) adsorption applications.

## 3. Results and Discussion

### 3.1. Morphological and Structural Characteristics of Hollow Carbon Microspheres

**Figure 2:**
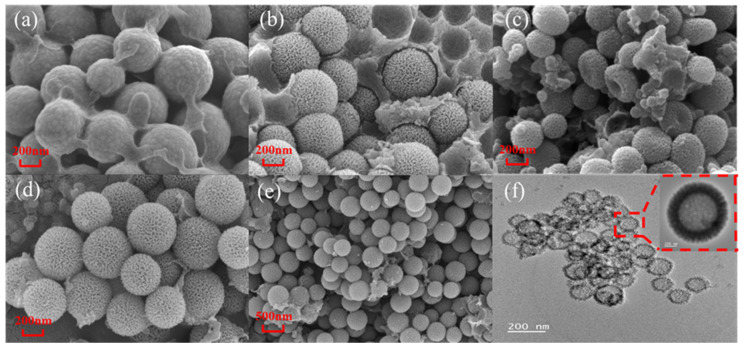
SEM images of SiO_2_@RF (**a**), HCM (**b**), HCM_5_ (**c**) and HCM_2.5_ (**d**,**e**), and TEM image of HCM_2.5_ (**f**).

Figure 2 illustrates the morphological evolution from SiO_2_@RF precursors to hollow carbon microspheres (HCM, HCM_5_ and HCM_2.5_) synthesized under different conditions, respectively. The data show that SiO_2_@RF, HCM, and HCM_2.5_ all exhibit relatively uniform spherical morphologies. Specifically, as shown in Figure 2a, the SiO_2_@RF have smooth surfaces but obvious interparticle adhesion. After template removal and subsequent activation treatment, a large amount of residual amorphous carbon matrix exists in HCM (Figure 2b), where microspheres are embedded within the matrix. After activation with KOH at different ratios (Figure 2c–f), HCM_2.5_ exhibits superior structural integrity and good monodispersity. It possesses uniform particle sizes of approximately 200–300 nm and a shell thickness of 40–60 nm, with abundant porous structures on the surface and no obvious adhesion or structural collapse. TEM images (Figure 2f) further confirm the uniform core–shell structure and porous carbon shell morphology. In comparison, HCM_5_ suffers from severe structural deformation and collapse owing to excessive activation, indicating that the preparation conditions for HCM_2.5_ are more favorable for maintaining the structural stability of hollow carbon microspheres.

EDS analysis in Table 1 shows significant differences in the C/O mass ratio among the three samples, which follows the order: HCM (86.80 wt% C) > HCM_5_ (82.62 wt% C) > HCM_2.5_ (80.93 wt% C). Consequently, HCM_2.5_ has the highest oxygen content of 19.07 wt%. The oxygen-containing functional groups can enhance the adsorption of Cr(VI) anions, including HCrO_4_^−^ and Cr_2_O_7_^2−^, through electrostatic interaction, and act as active sites for Cr(VI) reduction. Furthermore, the surface characteristics of the HCM_2.5_ exhibit a highly rough and porous texture, resembling a wrinkled or honeycomb-like structure. This unique morphology could provide abundant active sites for adsorption, thereby enhancing the potential applications of these materials in environmental remediation.

Furthermore, the pore structures of hollow carbon microspheres (HCM, HCM_5_ and HCM_2.5_) before and after activation were systematically characterized, and the results are presented in Figures S1–S3 and Table 2.

As shown in Table 2, it can be seen that after KOH activation, the BET specific surface area of HCM_2.5_ is significantly increased from 740.2 m^2^/g of HCM to 2165.0 m^2^/g, and the pore volume is enhanced from 1.179 cc/g to 2.231 cc/g. Meanwhile, the average pore size decreases from 3.19 nm to 2.06 nm, and the most probable pore diameter reduces from 8.60 nm to 3.89 nm, confirming that HCM_2.5_ possesses a more developed micro/mesoporous structure and higher pore density.

Figures S1–S3 display the N_2_ adsorption–desorption isotherms and pore size distributions of HCM, HCM_2.5_ and HCM_5_, respectively. The isotherms of both materials follow the characteristics of IUPAC type I + type IV composite curves, verifying their hierarchical micro-mesoporous structure. Compared with HCM, both HCM_2.5_ and HCM_5_ exhibit much steeper increases in adsorption capacity at low relative pressure (*P*/*P*_0_ < 0.1), indicating substantial increases in the number of micropores and strong micropore filling effects. Notably, HCM_5_ shows the most pronounced micropore filling behavior, consistent with its highest micropore content as reflected by its narrowest most probable pore diameter of 2.28 nm. In the medium-to-high relative pressure region, both activated samples show distinct hysteresis loops and continuous rises in adsorption capacity, demonstrating that the mesoporous structure is well retained. However, the hysteresis loop of HCM_5_ is narrower and the rise in adsorption capacity is less pronounced than that of HCM_2.5_, suggesting that excessive activation may have partially damaged or narrowed the pore channels. This is consistent with the severe structural deformation, fragmentation, and collapse of the microsphere framework observed for HCM_5_ in Figure 2c. Although activation introduces a large number of micropores, excessive etching destroys the interconnected pore network.

Therefore, KOH activation plays a pivotal role in precisely regulating the pore structure of carbon microspheres. It not only significantly improves the specific surface area and pore volume, but also promotes the development of a hierarchical pore architecture, predominantly microporous with contributions from mesopores. The resulting structure could provide abundant highly active sites for Cr(VI) adsorption, which is the key structural basis for the enhanced adsorption performance of HCM_2.5_, and moderate KOH activation (i.e., HCM_2.5_) is therefore the key to balancing structural integrity, surface functionality, and hierarchical porosity, rendering it the optimal candidate material for Cr(VI) removal.

**Figure 3:**
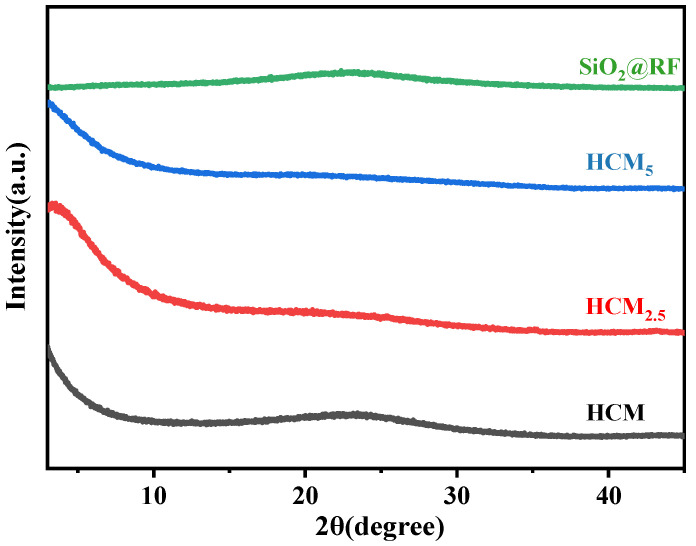
XRD patterns of SiO_2_@RF, HCM, HCM_5_ and HCM_2.5._

Figure 3 displays the XRD patterns of SiO_2_@RF, HCM, HCM_5_ and HCM_2.5_. No sharp crystalline diffraction peaks are observed in all samples, and only broad diffuse humps appear, indicating that all materials are of amorphous structure. All samples show a broad (0 0 2) peak at 2*θ* = 22°, confirming an amorphous carbon structure [35]. For HCM_2.5_ and HCM_5_, the original characteristic carbon peaks are further broadened with markedly reduced intensity, especially the (0 0 2) diffraction peak, which almost disappears. This is a typical manifestation that activation-induced pore formation destroys the locally ordered structure of carbon. It suggests that KOH activation etches the carbon skeleton and increases structural disorder and defects.

The significantly increased diffraction intensity in the low-angle region 2*θ* < 10° is a typical XRD feature of porous materials, proving that abundant micropores and mesopores are introduced after activation [35]. Consequently, the porosity is greatly improved and the amorphous degree is further enhanced. These findings are well consistent with the high porosity and high defect density characteristics of HCM_2.5_ and HCM_5_ obtained from BET characterization (Table 2), which collectively confirm the structural regulation effect of activation treatment. However, the amorphous carbon peaks of HCM_5_ disappear more completely and exhibit the highest structural disorder in comparison with HCM_2.5_. A higher alkali–carbon ratio further etches the carbon skeleton and continuously increases the number of micropores. Nevertheless, excessive activation may cause partial collapse of pore walls, thus reducing the specific surface area, which is slightly lower than that of HCM_2.5_. In addition, excessively small pore sizes may limit the diffusion of Cr(VI) ions; therefore, its actual adsorption performance is inferior to that of HCM_2.5_.

**Figure 4:**
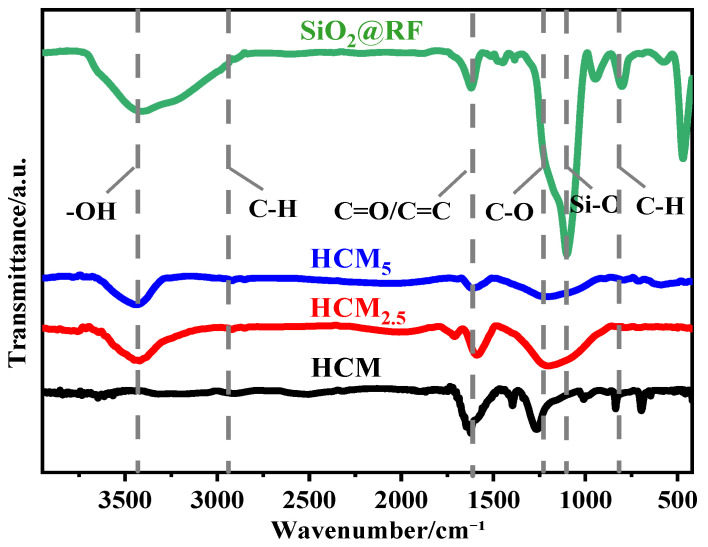
FT-IR cures of SiO_2_@RF, HCM, HCM_5_ and HCM_2.5._

**Figure 5:**
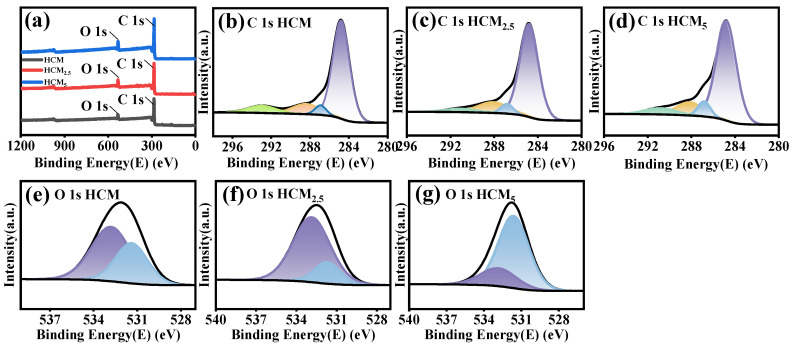
XPS spectra of HCM, HCM_5_ and HCM_2.5_: (**a**) Total XPS spectrum, (**b**) C 1s of HCM, (**c**) C 1s of HCM_2.5_, (**d**) C 1s of HCM_5_, (**e**) O 1s of HCM, (**f**) O 1s of HCM_2.5_, (**g**) O 1s of HCM_5._

To probe the effects mentioned above, FT-IR and XPS characterizations were further conducted on SiO_2_@RF, HCM, HCM_5_ and HCM_2.5_, with results shown in Figure 4 and Figure 5.

As illustrated in Figure 4, the Fourier transform infrared (FT-IR) spectra clearly reveal the structural evolution of the materials from the precursor to the activated carbon microspheres. For the SiO_2_@RF precursor, distinct characteristic absorption bands of hydroxyl groups (-OH) at approximately 3400 cm^−1^ and Si-O-Si bonds at approximately 1080 cm^−1^ are observed, which jointly confirm the successful synthesis of the core–shell structured precursor.

After carbonization and etching, the complete disappearance of the Si-O-Si characteristic peak in the FT-IR spectrum of HCM directly verifies the thorough removal of the silica template, while the carbon framework remains intact. This confirms the successful structural transformation from the core–shell precursor to hollow carbon microspheres. Moreover, this phenomenon provides direct structural evidence for elucidating the nature of the interactions between the core and shell components during the subsequent activation process.

A comparison of the samples with different activation degrees shows the following. Upon KOH activation, the intensities of the characteristic peaks corresponding to -OH, C=O, and C-O groups in the spectrum of HCM_2.5_ and HCM_5_ are significantly enhanced, forming abundant absorption bands in the 1500–1000 cm^−1^ region [36]. Meanwhile, the stretching vibration peak of the aromatic C=C bond is still clearly retained, indicating that the activation process does not damage the main carbon framework but instead induces targeted surface modification. This modification introduces a high density of oxygen-containing functional groups, which provide abundant active sites for Cr(VI) adsorption and lay a structural foundation for its excellent adsorption performance [37]. In contrast, the enhancement in the intensities of the oxygen-containing functional group peaks for HCM_5_ is weaker than that for HCM_2.5_. This indicates that excessive oxidation consumes surface active sites and triggers the desorption and decomposition of oxygen-containing functional groups at high temperatures, ultimately reducing the efficiency of introducing these functional groups.

Moreover, as shown in Figure 5a, the full XPS survey spectra of HCM, HCM_5_ and HCM_2.5_ only exhibit characteristic C 1s (~285 eV) and O 1s (~532 eV) peaks, confirming the high purity of the carbon microspheres. Notably, the O 1s peak intensities of HCM_2.5_ and HCM_5_ are significantly higher than that of HCM, indicating a remarkable increase in surface oxygen content after KOH activation. The high-resolution C 1s spectra (Figure 5b–d) of both samples are deconvoluted into four components: C-C/C-H (~284.8 eV) (purple peak), C-O (~286.3 eV) (blue peak), π-π* (~293.5 eV) (green peak) and O=C-O (~288.8 eV) (orange peak) [38]. The dominant C-C/C-H peak and π-π* peak confirm the intact carbon skeleton of HCM_2.5_ and HCM_5_ after activation, while the obviously increased proportion of C-O and O=C-O components verifies the successful introduction of abundant oxygen-containing functional groups [39].

For the O 1s spectra (Figure 5e–g), the purple fitted peak is assigned to C-O functional groups at approximately 533.0 eV, and the blue fitted peak corresponds to C=O functional groups at around 531.7 eV [35]. In acidic solution systems, C-O groups mainly combine Cr(VI) anions through hydrogen bonding and improve surface mass transfer efficiency, while C=O functional groups serve as electron donors to realize the reduction of Cr(VI) to Cr(III) and immobilize the generated Cr(III) via electrostatic interaction. In terms of peak area, pristine HCM contains low contents of both functional groups, lacking sufficient polar binding sites and reductive active sites, and thus displays the worst Cr(VI) adsorption performance. As for activated HCM_2.5_, its peak area of C-O functional groups is the largest among the three samples, and it also maintains a moderate proportion of C=O groups, forming an optimal functional group combination dominated by high-content C-O and assisted by appropriate C=O. This structure not only ensures the effective binding of Cr(VI) on the material surface and its efficient diffusion toward internal active sites, but also achieves the reduction and stable immobilization of Cr(VI) with the aid of C=O groups, hence delivering the optimal adsorption performance.

Although HCM_5_ has a further increased proportion of C=O functional groups, its peak area of C-O groups decreases significantly, which leads to insufficient surface polarity and the increased diffusion resistance of Cr(VI). Moreover, the reduced Cr(III) cannot be well fixed due to the lack of adequate polar sites, so that abundant high-activity C=O sites fail to be fully utilized, resulting in poorer adsorption performance than HCM_2.5_. In summary, the excellent Cr(VI) adsorption capacity of HCM_2.5_ originates not from the advantage of a single functional group content, but from the synergistic effect between C-O and C=O functional groups.

### 3.2. Adsorption Performance of HCM_2.5_ for Cr(VI)

**Figure 6:**
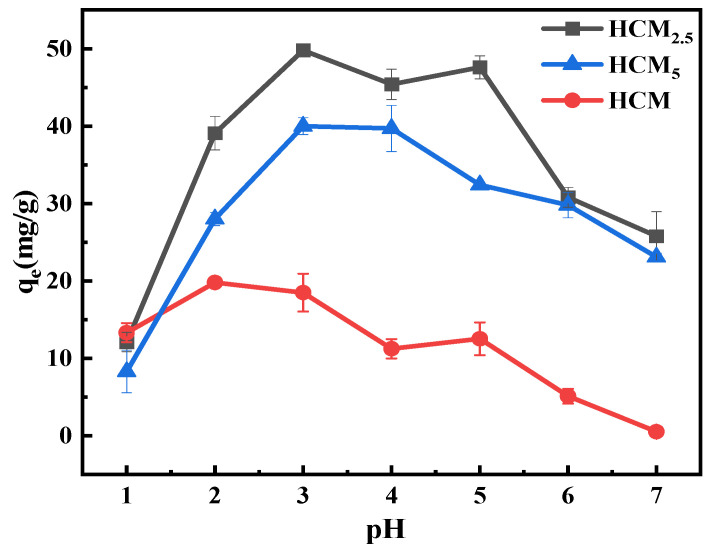
Adsorption capacities of HCM, HCM_2.5_, HCM_5_ for 50 ppm Cr(VI) at different pH.

The effect of solution pH on the Cr(VI) adsorption performance of HCM, HCM_2.5_, and HCM_5_ was investigated, and the results are shown in Figure 6. All three samples exhibit typical pH-dependent adsorption behavior: their adsorption capacities first increase and then decrease with increasing pH, reaching a maximum at pH 3.

Notably, HCM_2.5_ consistently shows the highest adsorption capacity across the entire tested pH range, achieving 49.8 mg/g at pH 3, which is significantly higher than those of HCM_5_ (~40 mg/g) and HCM (~20 mg/g). The superior performance of HCM_2.5_ can be attributed to its hierarchical micro-mesoporous structure with a high specific surface area (2165.0 m^2^/g) and abundant oxygen-containing functional groups (19.07 wt%), which not only provide sufficient adsorption sites but also enable strong electrostatic interactions with HCrO_4_^−^/Cr_2_O_7_^2−^.

In contrast, although HCM_5_ also possesses a high specific surface area and oxygen content, the impaired pore connectivity due to excessive activation limits the accessibility of its active sites, resulting in lower adsorption capacity and poorer pH stability. The pristine HCM, with its low specific surface area and undeveloped pore structure, exhibits the weakest adsorption performance and almost loses its effectiveness under neutral conditions. These results further confirm that moderate KOH activation is critical for balancing structural integrity, surface functionality, and porous structure, making HCM_2.5_ the optimal candidate adsorbent for Cr(VI) removal over a wide pH range. The carbon microspheres synthesized with an alkali-to-carbon ratio of 2.5 were selected as the main adsorbent in this study, abbreviated as HCM_2.5_.

**Figure 7:**
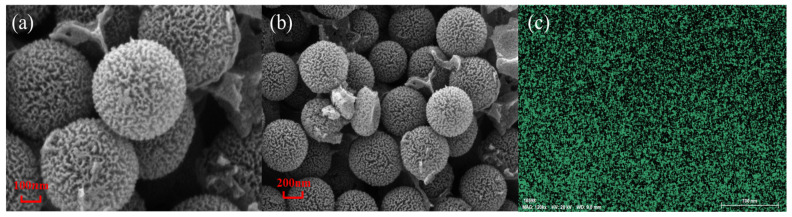
(**a**,**b**) SEM image of HCM_2.5_ after Cr(VI) adsorption (HCM_2.5_-Cr), (**c**) Cr distribution of HCM_2.5_-Cr analyzed by EDS.

Figure 7a,b shows the morphology of HCM_2.5_ after Cr(VI) adsorption (HCM_2.5_-Cr). The images show that, similar to the pristine carbon microspheres (Figure 2c), HCM_2.5_-Cr preserves a well-defined spherical shape with a rough, porous and wrinkled surface and good monodispersity. This confirms that the original morphology of the carbon microspheres is well preserved before and after adsorption, indicating excellent structural stability and reusability, further confirming the robustness of the structure. Further, such robustness is advantageous for applications requiring durable and reliable performance under varying environmental conditions. Figure 7c demonstrates that Cr(VI) is uniformly adsorbed onto the surface of HCM_2.5_ carbon microspheres.

Based on the above characterization results, it can be concluded that KOH activation simultaneously optimizes the pore structure and modifies the surface functional groups of the carbon microspheres, thereby fundamentally enhancing the adsorption performance of the material toward Cr(VI). To further reveal the adsorption mechanism, FTIR and XPS were employed on HCM_2.5_ and HCM_2.5_-Cr.

**Figure 8:**
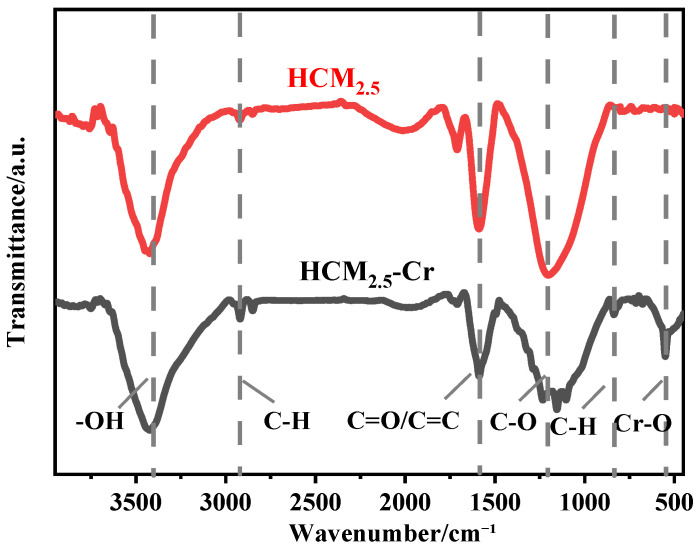
FTIR curves of HCM_2.5_ and HCM_2.5_-Cr before and after Cr(VI) adsorption.

As shown in Figure 8, HCM_2.5_ displays characteristic absorption peaks at ~3400 cm^−1^ (-OH), ~1600 cm^−1^ (C=O/C=C) and ~1100 cm^−1^ (C-O), indicating abundant oxygen-containing functional groups on the surface of HCM_2.5_. The characteristic aromatic C=C stretching peak at ~1600 cm^−1^ and the C-H stretching peak at ~2900 cm^−1^ are fully retained after Cr(VI) adsorption, which demonstrates that the adsorption process does not destroy the integrity of the carbon matrix, confirming the excellent structural stability of the material [40]. After Cr(VI) adsorption, the peak intensities of HCM_2.5_-Cr in the above regions are significantly enhanced, and a new characteristic Cr-O peak appears at ~500–600 cm^−1^, demonstrating that Cr(VI) interacts with surface-oxygen-containing functional groups (hydroxyl, carbonyl, and carboxyl, etc.) via coordination/complexation and forms stable Cr-O chemical bonds [41].

**Figure 9:**
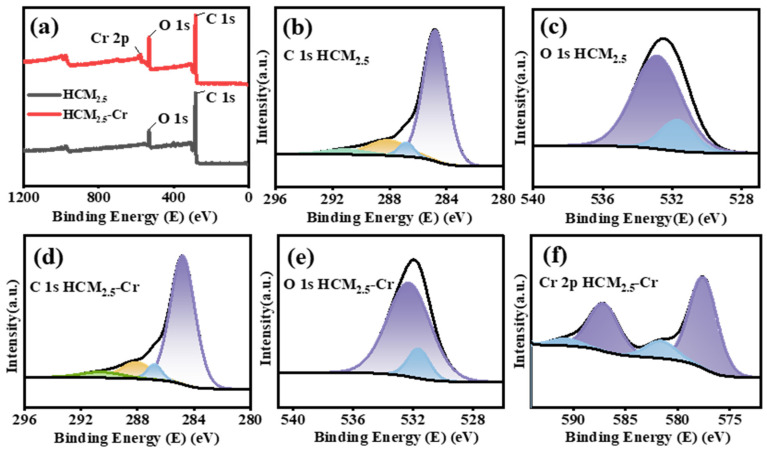
XPS spectra of HCM_2.5_ and HCM_2.5_-Cr: (**a**) Total XPS spectrum, (**b**) C 1s of HCM_2.5_, (**c**) O 1s of HCM_2.5_, (**d**) C 1s of HCM_2.5_-Cr, (**e**) O 1s of HCM_2.5_-Cr, (**f**) Cr 2p of HCM_2.5_-Cr.

Additionally, as shown in Figure 9a, the full XPS survey spectra of HCM_2.5_ only exhibit characteristic C 1s and O 1s peaks, while a new Cr 2p peak emerges after Cr adsorption, confirming the successful immobilization of Cr onto the surface of the carbon microspheres. The high-resolution C 1s spectra (Figure 9b,d) reveal a significant increase in the peak area proportions of the C-O (blue peak), O-C=O (orange peak), and π→π* (green peak) components after adsorption, indicating that oxygen-containing functional groups and aromatic conjugated structures are the core active sites for Cr(VI) adsorption. The changes in the relative intensities of C=O (blue peak) and C-O (purple peak) in the O 1s spectra (Figure 9c,e) further verify the interaction between these functional groups and Cr. The deconvolution results of the Cr 2p spectrum (Figure 9f) demonstrate that Cr exists predominantly in the form of Cr(III) after adsorption, proving that HCM_2.5_ not only adsorbs Cr(VI) but also reduces it to low-toxicity Cr(III), achieving the functions of efficient adsorption and detoxification. Specifically, the high-resolution Cr 2p spectrum can be deconvoluted into two sets of characteristic peaks. The purple peaks at ~577.8 eV (Cr 2p_3/2_) and ~587.2 eV (Cr 2p_1/2_) correspond to the typical orbital signals of Cr(III), while the weak blue peaks at ~580.0 eV (Cr 2p_3/2_) and ~590.0 eV (Cr 2p_1/2_) are assigned to Cr(VI). Building on the XPS results, combined with FTIR, BET, and XRD analyses, a complete structure–performance relationship is established for the KOH-activated carbon microspheres. Collectively, these findings synergistically demonstrate that KOH activation simultaneously optimizes the pore structure and modifies the surface functional groups of the carbon microspheres, constructing a multi-mechanism adsorption framework that includes physical adsorption, chemical complexation, and reductive detoxification, which fundamentally enhances the adsorption performance of the HCM_2.5_ toward Cr(VI) [42].

### 3.3. Effects of pH and Dosage on Adsorption Performance

The pH is a critical factor influencing the adsorption of metal ions, as it directly determines the speciation of ions in aqueous solution. To elucidate the adsorption mechanism and performance of HCM_2.5_, the effect of pH on the surface charge of the material was systematically investigated.

**Figure 10:**
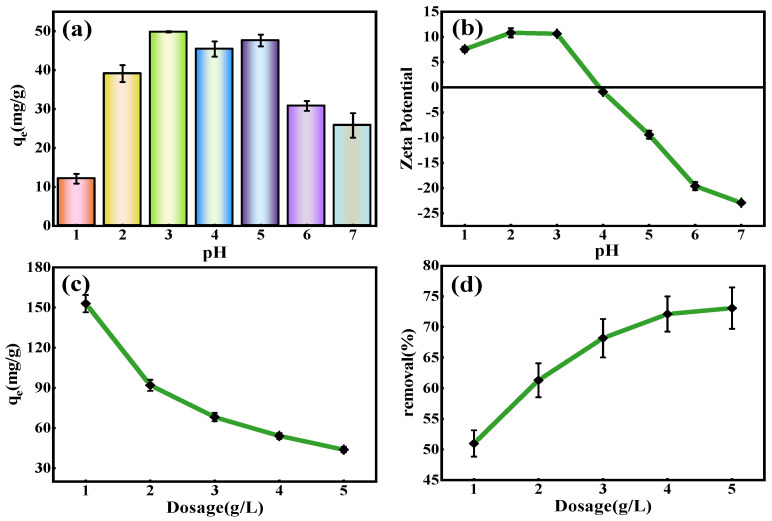
(**a**) For the adsorption experiment with an initial Cr(VI) concentration of 50 ppm at pH values ranging from 1 to 7 and a dosage of 1 g/L, (**b**) the Zeta potential data of HCM_2.5_, (**c**,**d**) the effects of the dosage of HCM_2.5_ at 300 ppm Cr(VI) (pH = 3) on the adsorption capacity and removal rate.

Figure 10a presents the adsorption behavior of Cr(VI) by HCM_2.5_ at different pH values. The adsorption capacity reaches the maximum at pH = 3, with a removal rate of 99.6%. Zeta potential measurements (Figure 10b) reveal that the isoelectric point of HCM_2.5_ is 4.0, and its potential gradually decreases with increasing pH. Generally, under acidic conditions (pH = 2–6.8), Cr(VI) exists mainly as HCrO_4_^−^/Cr_2_O_7_^2−^ anions [43]. At pH < 4.0, the material surface of HCM_2.5_ is positively charged, enabling efficient capture of Cr(VI) anions via electrostatic attraction. The maximum adsorption capacity is 49.8 mg/g with a removal efficiency of 99.6% at pH 3. However, at pH 5, the contribution of electrostatic adsorption decreases significantly, while chemical complexation/coordination becomes dominant; meanwhile, the ultrahigh specific surface area provides abundant physical adsorption sites, maintaining a stable adsorption capacity, even with weakened electrostatic interaction at pH 5. At pH > 5.0, electrostatic repulsion between the material surface and Cr(VI) anions leads to a pronounced reduction in adsorption capacity.

Subsequently, the effect of adsorbent dosage on metal ion removal efficiency was studied at the optimal pH (pH = 3.0). Figure 10c,d displays the adsorption results of HCM_2.5_ in 300 ppm Cr(VI) solution (pH = 3) with varying dosages. The removal efficiency of Cr(VI) increases gradually with rising adsorbent dosage, whereas the unit adsorption capacity decreases accordingly. The optimal dosage of HCM_2.5_ is 5 g/L in 300 ppm Cr(VI) solution, achieving the highest removal efficiency of 73.08%.

### 3.4. Adsorption Kinetics and Thermodynamics

**Figure 11:**
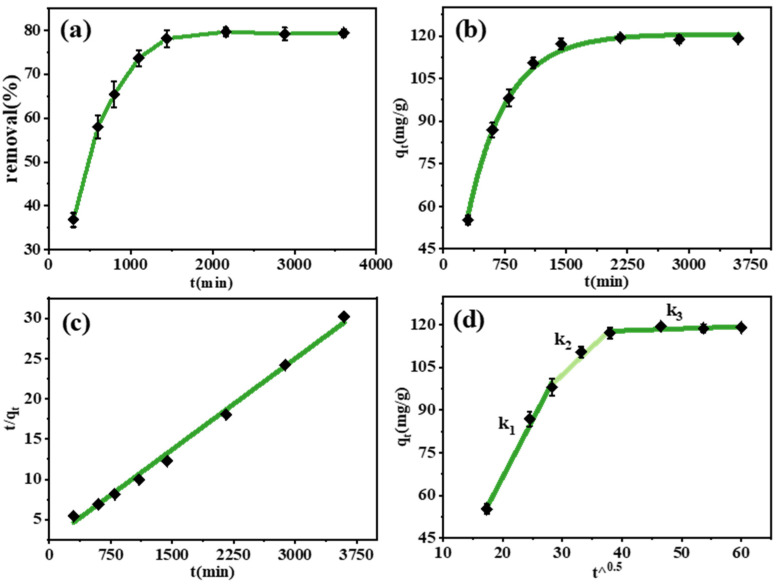
(**a**) The influence of reaction time on removal rate; (**b**) first-order kinetic fitting; (**c**) second-order kinetic fitting; (**d**) particle internal diffusion model fitting.

To investigate the adsorption and desorption mechanisms, kinetic experiments were conducted and the results are shown in Figure 11, Table 3 and Table 4. The initial concentration of Cr(VI) was set at 150 mg/L, with the adsorbent dosage of 1 g/L and the adsorption temperature of 25 °C, pH = 3. The adsorption capacity of HCM_2.5_ was measured at 300, 600, 800, 1100, 1400, 2160, 2880, and 3600 min. The adsorption kinetic data of HCM_2.5_ on Cr(VI) were fitted using the pseudo-first-order kinetic model (Equation (2)), pseudo-second-order kinetic model (Equation (3)), and intraparticle diffusion model (Equation (4)), respectively [44].

(2) $$\ln(q_{e}-q_{t})=\ln q_{e}-k_{1}t$$

(3) $$\frac{t}{q_{t}}=\frac{1}{k_{2}q_{e}^{2}}+\frac{t}{q_{e}}$$

(4) $$q_{t}=k_{id}t^{0.5}+C$$

where *q_e_* represents the Cr(VI) adsorption amount at the adsorption equilibrium moment (mg/g); *q_t_* represents the Cr(VI) adsorption amount at time *t* (mg/g); *t* is the adsorption time (min); *k*_1_ is the rate constant of the pseudo-first-order kinetics (min^−1^); *k*_2_ is the rate constant of the pseudo-second-order kinetics (g^−1^·min^−1^); *k_id_* is the rate constant of the intraparticle diffusion model (mg/(g·min^0.5^)); and *C* corresponds to the boundary layer effect parameter.

Figure 11a shows that the Cr(VI) adsorption capacity increases with the prolongation of reaction time. A rapid increase in adsorption capacity occurs within the initial stage of 0–1400 min, which can be attributed to the rapid binding of Cr(VI) ions to abundant unoccupied adsorption sites on the material surface of HCM_2.5_. The adsorption process reaches equilibrium after approximately 24 h.

Furthermore, as can be seen from Table 3 and Figure 11b,c, the maximum adsorption capacities calculated by the pseudo-first-order and pseudo-second-order kinetic models are 120.6 mg/g and 132.8 mg/g, respectively. The theoretical equilibrium adsorption capacity (*q_e_* = 120.6 mg/g) calculated by the pseudo-first-order model is closer to the experimental value, with a higher coefficient of determination *R*^2^. These results further indicate that the adsorption behavior of HCM_2.5_ toward Cr(VI) is dominated by physical adsorption or physicochemical interactions, including electrostatic attraction, pore diffusion, and surface complexation.

The intraparticle diffusion model (Figure 11d) exhibits a distinct three-stage linear profile without passing through the origin, confirming that the adsorption rate is jointly controlled by film diffusion and intraparticle diffusion, rather than a single rate-limiting step. The first stage with the steepest slope corresponds to the film diffusion (external diffusion) process: driven by a high concentration gradient, Cr(VI) ions rapidly diffuse from the bulk solution to the adsorbent surface, and the adsorption rate is dominated by film diffusion. The second stage with a moderate slope represents the synergistic control of film diffusion and intraparticle diffusion: as the surface active sites are gradually occupied, and Cr(VI) ions diffuse into the internal pores of the material, the adsorption rate is co-governed by both diffusion processes. The third stage with an almost flat slope is the adsorption equilibrium stage, where the active sites are fully saturated, the mass transfer resistance reaches its maximum, and the adsorption capacity remains essentially constant.

**Figure 12:**
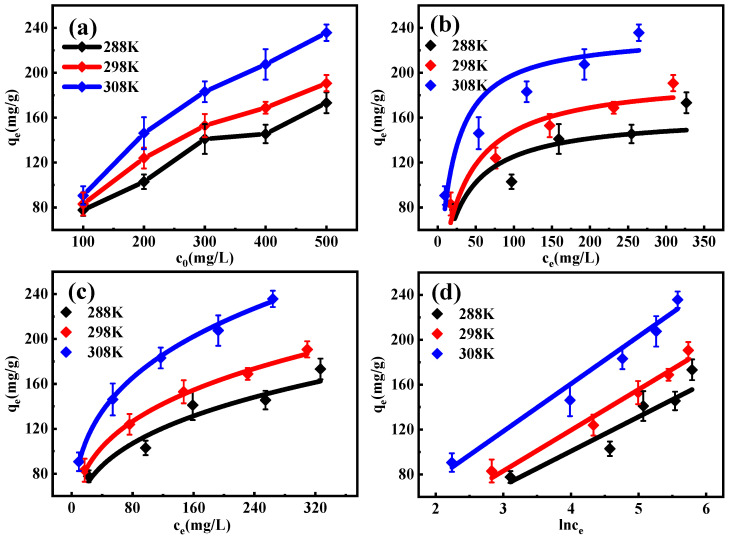
(**a**) Equilibrium adsorption data for Cr(VI) on HCM_2.5_ at different temperature and different initial concentration (*c*_0_). The fitting curves of the Langmuir model (**b**), Freundlich model (**c**), and Temkin isotherms model (**d**) for the adsorption of Cr(VI).

The adsorption behavior of HCM_2.5_ at 288.15 K, 298.15 K, and 308.15 K was investigated using Langmuir (Equation (5)), Freundlich (Equation (6)), and Temkin (Equation (7)) models, and the equilibrium constants along with maximum adsorption capacities were calculated [44,45,46]. The adsorption isotherm experimental conditions were set as follows: pH = 3, a Cr(VI) concentration varying from 100 to 500 mg/L, and an adsorption duration of 24 h. The results are shown in Figure 12 and Table 5 and Table 6.

(5) $$q_{e}=\frac{K_{L}q_{\max}c_{e}}{1+K_{L}c_{e}}$$

(6) $$q_{e}=K_{F}c_{e}^{\frac{1}{n}}$$

(7) $$q_{e}=\left(\frac{RT}{b}\right)\ln\left(K_{T}c_{e}\right)$$

where *q_e_* denotes the equilibrium adsorption capacity of Cr(VI) (mg/g); *c_e_* represents the equilibrium concentration of Cr(VI) (mg/L); *q_max_* signifies the maximum adsorption capacity of Cr(VI) under monolayer adsorption conditions as per the Langmuir model (mg/g); *K_F_* corresponds to the Freundlich constant; *K_L_* is the equilibrium constant of the Langmuir isotherm; *n* is a dimensionless constant reflecting adsorption intensity; *b* stands for the conformational potential parameter; and *K_T_* corresponds to the Temkin constant.

The results demonstrate that the Freundlich model exhibits the best-fitting performance among the three models, with the highest correlation coefficient *R*^2^. The Freundlich isotherm is an empirical model suitable for heterogeneous surfaces and multilayer adsorption, which indicates that the adsorption of Cr(VI) onto HCM_2.5_ occurs on a heterogeneous surface, dominated by a synergistic mechanism of monolayer and multilayer adsorption. The Freundlich constant *K_F_* increases with the increase of temperature, further confirming the endothermic nature of the adsorption process. Meanwhile, the 1/*n* values of all temperature groups are less than 1, proving that the adsorption is favorable adsorption. The good fitting of the Freundlich model is consistent with the pore structure and abundant surface functional groups of HCM_2.5_, which provide sufficient heterogeneous active sites for the adsorption of Cr(VI).

To better understand the mechanism of the adsorption process, the changes in Gibbs free energy (Δ*G*, kJ/mol), enthalpy of adsorption (Δ*H*, kJ/mol), and entropy of adsorption (Δ*S*, kJ/(K·mol)) were calculated using the following equations:

(8) $$\ln q_{m}=\frac{\Delta S}{R}-\frac{\Delta H}{RT}$$

(9) $$\Delta G=-RT\ln q_{m}$$

where *q_m_* is the maximum adsorption capacity calculated using the Langmuir model; Δ*S* is the entropy change during adsorption (J/(mol·K)); Δ*H* is the enthalpy change during adsorption (kJ/mol); *R* is ideal gas constant, 8.314 J/(mol·K); and Δ*G* is the Gibbs free energy change during adsorption (kJ/mol).

The thermodynamic parameters Δ*S*, Δ*G*, and Δ*H* for the adsorption of Cr(VI) by HCM_2.5_ were calculated, and the results are presented in Table 6. Δ*S* and Δ*H* were determined from the intercept and slope of the linear fitting plot (Figure S3). Within the temperature range of 288.15 K to 308.15 K, the calculated Δ*H* = 13.78 kJ/mol (Δ*H* > 0) indicates that the adsorption process is endothermic. Meanwhile, the negative Δ*G* values obtained at all tested temperatures suggest that the adsorption of Cr(VI) onto HCM_2.5_ is thermodynamically spontaneous. The positive Δ*S* value implies an increase in the degree of disorder at the solid–liquid interface during adsorption [47]. Overall, thermodynamic analysis demonstrates that the adsorption of Cr(VI) by HCM_2.5_ is more favorable at elevated temperatures.

Table 7 compares the thermodynamic parameters of HCM_2.5_ with other Cr(VI) adsorbents at 298.15 K. HCM_2.5_ shows a more negative Δ*G* (−13.09 kJ·mol^−1^), indicating stronger spontaneous adsorption at room temperature. The Δ*S* value is 90.18 J·mol^−1^·K^−1^, notably higher than those of most reported materials, corresponding to an entropy-increasing process that further confirms the spontaneity of adsorption and suggests strong interactions between the adsorbent surface and Cr(VI) species. The moderate positive Δ*H* (13.78 kJ·mol^−1^) reflects an endothermic nature, consistent with improved uptake at higher temperatures.

Furthermore, HCM_2.5_ is nearly insoluble in acidic environments, exhibits excellent solid–liquid separation performance with no secondary pollution, and exhibits a higher adsorption capacity than similar materials, with a maximum adsorption capacity with a *q*_max_ value of 235.7 mg/g (as shown in Table 8).

### 3.5. Influence of Other Ions

**Figure 13:**
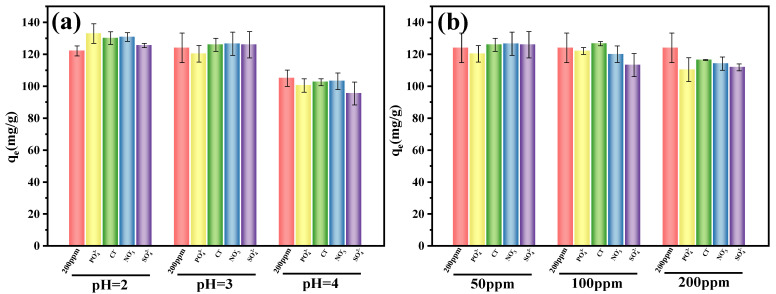
(**a**) Effects of coexisting anions on the Cr(VI) adsorption capacity of HCM_2.5_ under different pH values at an initial concentration of 50 ppm, (**b**) effects of coexisting anions on the Cr(VI) adsorption capacity of HCM_2.5_ under different initial concentrations at pH = 3_._

Considering that acidic wastewater may contain a large number of coexisting ions such as sulfate (SO_4_^2−^), nitrate (NO_3_^−^), chloride (Cl^−^) and phosphate (PO_4_^3−^), this study further investigated the influence of these ions on the Cr(VI) adsorption process by HCM_2.5_. As shown in Figure 13, the initial concentration of Cr(VI) was set at 200 mg L^−1^, and the concentration of other interfering ions was 50, 100, and 200 mg L^−1^. The solution pH was adjusted to 2, 3, and 4 with dilute hydrochloric acid, the adsorbent dosage was 1 g L^−1^, and the contact time was 24 h.

As shown in Figure 13a, the effects of coexisting anions on the Cr(VI) adsorption performance of HCM_2.5_ were investigated under different pH conditions. The presence of Cl^−^, NO_3_^−^, and SO_4_^2−^ exerts no significant inhibitory effect on the Cr(VI) adsorption capacity of HCM_2.5_ over the pH range of 2–4, and even leads to a slight enhancement under certain conditions. This phenomenon can be attributed to the electric double-layer compression effect: high concentrations of coexisting anions in solution compress the electric double layer at the adsorbent surface, weakening the electrostatic repulsion between the positively charged adsorbent and Cr(VI) anions (HCrO_4_^−^/Cr_2_O_7_^2−^), thereby facilitating Cr(VI) adsorption to a certain extent. As shown in Figure 13b, the effect of coexisting anions on Cr(VI) adsorption onto HCM_2.5_ was further investigated at different initial Cr(VI) concentrations (50, 100, and 200 ppm) at a fixed pH of 3. The results revealed that the adsorption capacity of HCM_2.5_ for Cr(VI) generally decreased with increasing initial Cr(VI) concentration, which can be attributed to the gradual saturation of available adsorption sites on the adsorbent surface and the increased mass transfer resistance caused by higher ionic strength at elevated concentrations. At the lowest initial concentration (50 ppm), the inhibitory effect of coexisting anions (Cl^−^, NO_3_^−^, and SO_4_^2−^) was negligible, as the abundant active sites on HCM_2.5_ were sufficient to accommodate both Cr(VI) and competing anions without significant interference. However, as the initial Cr(VI) concentration increased to 100 ppm and further to 200 ppm, the competitive adsorption effect of coexisting anions became progressively more pronounced.

In contrast, the influence of phosphate species is relatively complex. In the pH range of 2.15–7.20, phosphate mainly exists in the form of H_2_PO_4_^−^ [48]. It possesses a molecular size and spatial structure highly similar to those of HCrO_4_^−^ (the dominant Cr(VI) species). Both substances can form coordination bonds with active sites on the surface of HCM_2.5_, such as oxygen-containing functional groups and metal active sites, thus competing for available adsorption sites with Cr(VI). When the solution pH is lower than 2.15, phosphate primarily occurs as neutral H_3_PO_4_ molecules [48]. Such neutral molecules can scarcely compete with negatively charged Cr(VI) anions for positively charged adsorption sites on the adsorbent. Even trace amounts of ionized phosphate species show far lower charge density and coordination capacity than those formed at higher pH values, leading to negligible competitive effects. Accordingly, as displayed in Figure 13a,b, obvious inhibitory effects induced by phosphate are only detected at pH 3 and pH 4, and this suppression trend is further enhanced with the increase in initial solution concentration. At pH 2, the electric double-layer compression effect dominates. Hydrogen ions and other cations accumulate on the adsorbent surface to compress the electric double layer, which weakens the electrostatic repulsion between the adsorbent and Cr(VI) ions and accordingly achieves a slight promotion effect on Cr(VI) adsorption.

### 3.6. Cyclic Regeneration Experiment

**Figure 14:**
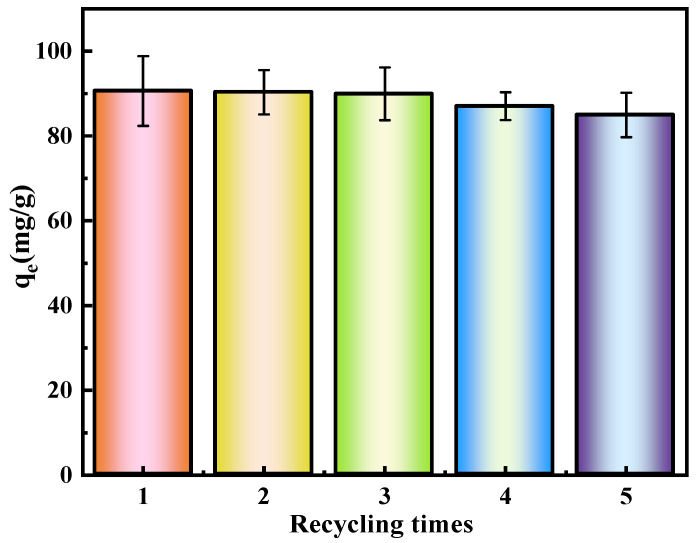
Five removal cycles of Cr(VI) by HCM_2.5_ in pH = 3 system.

The regeneration experiments demonstrate that HCM_2.5_ exhibits excellent cyclic stability and reusability. As shown in Figure 14 and the corresponding data, at pH = 3, the adsorption capacity of HCM_2.5_ slowly decreases from an initial value of 90.61 mg/g to 84.26 mg/g after five consecutive adsorption–desorption cycles for Cr(VI), retaining more than 93% of its initial capacity with only slight overall attenuation. Statistical analysis of the multi-cycle adsorption data shows that the standard deviation of *q*_e_ across cycles is only 3.28–8.24 mg/g, with fluctuations far smaller than the overall decline in average adsorption capacity. These results confirm that the material’s adsorption performance shows excellent repeatability and stability over repeated use, further supporting its superior cyclic performance.

The BET results (Table 9, Figures S5 and S6) show that after two adsorption–desorption cycles, the *S_BET_* of HCM_2.5_ decreased from 2165.0 m^2^/g to 1973.9 m^2^/g, and the pore volume decreased from 2.231 cm^3^/g to 1.916 cm^3^/g, corresponding to reductions of 8.8% and 14.1%, respectively. After the first cycle, the average pore size remained unchanged at 2.06 nm, while the most frequent pore diameter decreased from 3.89 nm to 3.57 nm, indicating a shift of the mesopore distribution toward smaller sizes. After the second cycle, the average pore size increased slightly, accompanied by a decrease in pore volume and a negligible change in the most frequent pore diameter. These observations suggest that a small fraction of micropores underwent blocking or structural collapse during cycling, while the majority of the pore structure remained intact after repeated use. Figure 7a,b also indicates that the spherical structure of the carbon microspheres remains intact before and after adsorption, which is consistent with the BET analysis results.

EDS results (Table 10) reveal that the C/O atomic ratio of HCM_2.5_ exhibits only a minor change from the initial 84.97/15.03 to 86.62/13.38 after the second cycle, indicating that the surface-oxygen-containing functional groups are largely preserved and can provide continuous active sites for chemical adsorption.

**Figure 15:**
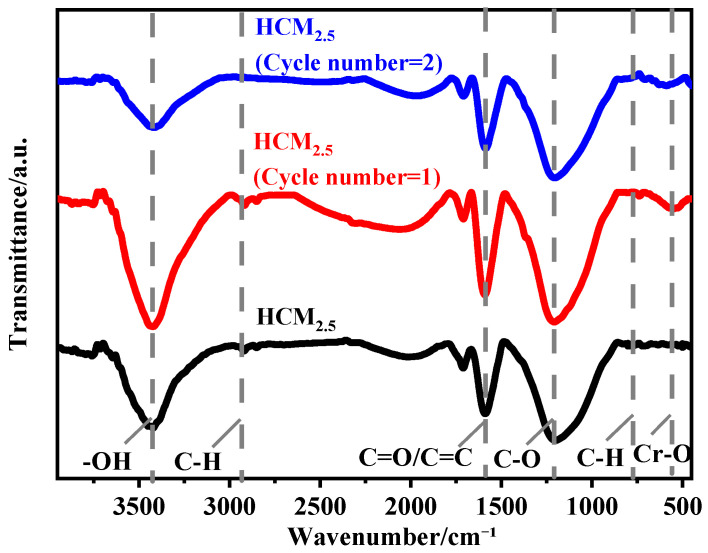
The FT-IR of HCM_2.5_, HCM_2.5_ (Cycle number = 1) and HCM_2.5_ (Cycle number = 2).

Comparative FT-IR spectra (Figure 15) of the pristine HCM_2.5_ and the regenerated HCM_2.5_ samples after one and two adsorption–desorption cycles show no significant attenuation in the intensities of characteristic peaks corresponding to oxygen-containing functional groups, including -OH (~3400 cm^−1^), C=O (~1600 cm^−1^), and C-O (~1100 cm^−1^). This confirms that the desorption process does not induce the loss of active sites, thereby ensuring the efficient restoration of adsorption performance. These findings synergistically verify that HCM_2.5_ maintains high stability in both structure and functional groups during the adsorption–regeneration process, showing great application prospects in the practical treatment of Cr(VI)-containing wastewater.

### 3.7. Adsorption Mechanism

**Figure 16:**
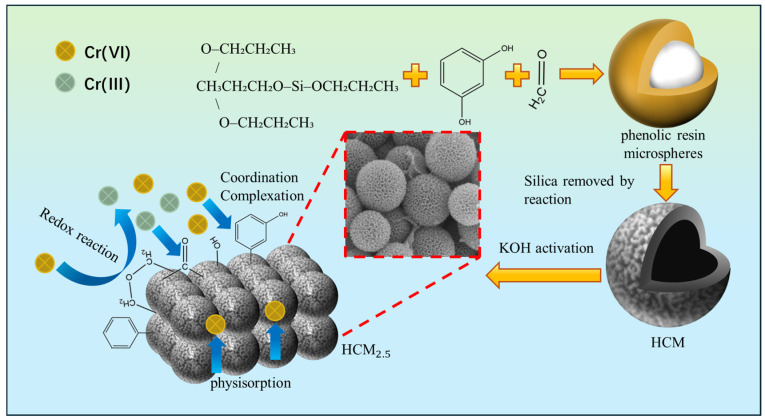
Multi-mechanism adsorption system of physisorption, chemical complexation and reductive detoxification.

The removal mechanism of Cr(VI) by HCM_2.5_ can be attributed to the enhanced physical adsorption provided by its ultrahigh specific surface area and abundant porous structure, together with the synergistic effect of chemical adsorption via interactions of surface functional groups. The adsorption mechanism of HCM_2.5_ for Cr(VI) is illustrated in Figure 16. KOH activation can simultaneously optimize the pore structure and modify the surface functional groups of the carbon microspheres. A large number of oxygen-containing functional groups such as hydroxyl (-OH) and carbonyl (C=O) are distributed on the surface of HCM_2.5_. Thereby, a multi-functional adsorption system is constructed: the abundant micro- and mesoporous structures endow the material with excellent physical adsorption capacity; electrostatic attraction and chemical complexation occur between surface active sites and Cr(VI) species. Meanwhile, electron-rich hydroxyl and other functional groups on the surface act as electron donors, reducing highly toxic Cr(VI) into low-toxicity and low-mobility Cr(III). The generated Cr(III) can be further chelated and immobilized by the negatively charged oxygen-containing groups on the material surface. The above synergistic multi-mechanism effect fundamentally enhances the adsorption performance of HCM_2.5_ toward Cr(VI). In addition, an acidic environment can further promote the binding interaction of Cr(VI), which mainly exists as HCrO_4_^−^/Cr_2_O_7_^2−^ under acidic conditions, with the adsorption sites.

## 4. Conclusions

A novel phenol–formaldehyde resin-derived carbon microsphere (HCM_2.5_) was designed and synthesized via a hard-template method combined with a KOH activation strategy. The obtained HCM_2.5_ can be used to effectively remove Cr(VI) from acidic wastewater. Characterization via XRD, SEM, BET, FTIR, and XPS revealed that HCM_2.5_ possesses a hollow porous structure with excellent surface properties, exhibiting a specific surface area of 2165 m^2^/g. A Cr(VI) removal efficiency exceeding 99.6% could be achieved in 50 ppm acidic solution, with excellent performance at pH 2–5, demonstrating superior structural stability and recyclability for multiple reuse cycles. The adsorption performance of the activated HCM_2.5_ is significantly superior to that of the unactivated HCM. Kinetic and thermodynamic analyses demonstrated that the adsorption of Cr(VI) by HCM_2.5_ is an endothermic process controlled by intra-particle diffusion, with the removal mechanism involving a combination of physical adsorption, chemical complexation, and reductive detoxification. In conclusion, HCM_2.5_ provides an effective solution for heavy metal removal from acidic wastewater and shows promising application prospects.

## Figures and Tables

**Table 1:** The ratio of C/O in HCM, HCM_5_ and HCM_2.5_ determined by EDS.

	C/O (*w*%)	C/O (Atom%)
HCM_5_	82.62/17.37	86.36/13.63
HCM_2.5_	80.93/19.07	84.97/15.03
HCM	86.80/13.20	89.76/10.24

**Table 2:** The pore structure characteristics of hollow carbon microspheres (HCM, HCM_5_ and HCM_2.5_) analyzed using BET method.

Samples	*S*_BET_ (m^2^/g)	Pore Volume (cc/g)	Average Pore Size (nm)	Most Frequent Pore Diameter (nm)
HCM_5_	2047.4	2.121	1.93	2.28
HCM_2.5_	2165.0	2.231	2.06	3.89
HCM	740.2	1.179	3.19	8.60

**Table 3:** Pseudo-first-order model and pseudo-second-order kinetic model parameters for adsorption of Cr(VI).

	Pseudo-First-Order	Pseudo-Second-Order
*q_e_*	120.6 mg/g	132.8 mg/g
*k*	0.0021 min^−1^	0.0002 g^−1^·min^−1^
*R* ^2^	0.9970	0.9943

**Table 4:** Parameters of intraparticle diffusion fitting model for adsorption of Cr(VI).

	*k_i_* (mg/(g·min^0.5^))		*C* (mg/g)	*R* ^2^
Stage I	*k*_1_	4.020	14.01	0.9939
Stage II	*k*_2_	1.979	43.04	0.9732
Stage III	*k*_3_	0.0723	115.1	0.9628

**Table 5:** The fitted parameters of the Langmuir, Freundlich and Temkin models.

	Langmuir Model			Freundlich Model			Temkin Model	
*T* (K)	*R* ^2^	*K_L_ ^a^*	*q_m_ ^b^*	*R* ^2^	*K_F_ ^c^*	*n*	*R* ^2^	*K_T_ ^d^*
288.15	0.8129	0.0340	162.3	0.9482	29.13	3.370	0.9037	30.68
298.15	0.9046	0.0300	197.1	0.9925	35.96	3.481	0.9705	36.32
308.15	0.9149	0.0534	235.5	0.9982	46.68	3.462	0.9789	42.35

*^a^* the unit of *K_L_* is L/mg; *^b^* the unit of *q_m_* is mg/g; *^c^* the unit of *K_F_* is (mg/g)·${\left(mg/L\right)}^{-\frac{1}{n}}$; *^d^* the unit of *K_T_* is L/mg.

**Table 6:** Thermodynamic data of the adsorption process for HCM_2.5_.

*T* (K)	*q_m_* (mg/g)	Δ*G* (kJ·mol^−1^)	Δ*S* (J·mol^−1^·K^−1^)	Δ*H* (kJ·mol^−1^)	*R* ^2^
288.15	162.3	−12.19	90.18	13.78	0.9999
298.15	197.1	−13.09			
308.15	235.5	−13.99			

**Table 7:** Comparison of thermodynamic parameters with other reported adsorbents at 298.15 K.

Material	Δ*G* (kJ·mol^−1^)	Δ*S* (J·mol^−1^·K^−1^)	Δ*H* (kJ·mol^−1^)	Reference
Ppy/MoS_2_	−4.16	0.2518	71.01	[11]
DACS-CA	−0.097	3.71	3.71	[9]
Ox-g-C3N4/Pani-NF	−2.669	80.988	22.055	[5]
HCM_2.5_	−13.09	90.18	13.78	This work

**Table 8:** Performance comparison of similar materials.

Material	Activating Agent	pH	*q_max_* (mg/g)	Reference
Fox nutshell	H_3_PO_4_	2	79.5	[28]
Longan seed	NaOH	2	169.49	[26]
Peanut shell	KOH	4	13.28	[25]
HCM_2.5_	KOH	3	235.7	This work

**Table 9:** The pore structure characteristics of hollow carbon microspheres (HCM_2.5_, HCM_2.5_ (Cycle number = 1) and HCM_2.5_ (Cycle number = 2)) analyzed using BET method.

Samples	*S_BET_* (m^2^/g)	Pore Volume (cc/g)	Average Pore Size (nm)	Most Frequent Pore Diameter (nm)
HCM_2.5_	2165.0	2.231	2.06	3.89
HCM_2.5_ (Cycle number = 1)	2091.8	2.158	2.06	3.57
HCM_2.5_ (Cycle number = 2)	1973.9	1.916	2.24	3.74

**Table 10:** The EDS results of HCM_2.5_, HCM_2.5_ (Cycle number = 1) and HCM_2.5_ (Cycle number = 2).

	C/O (*w*%)	C/O (Atom%)
HCM_2.5_	80.93/19.07	84.97/15.03
HCM_2.5_ (Cycle number = 1)	82.64/17.36	86.38/13.62
HCM_2.5_ (Cycle number = 2)	82.83/17.17	86. 54/13.46

## Data Availability

The original contributions presented in this study are included in the article. Further inquiries can be directed to the corresponding authors.

## Supplementary Materials

The following supporting information can be downloaded at: https://www.mdpi.com/article/10.3390/nano16110669/s1.
