# Supplementary material for: High-Specific-Surface-Area Hollow Carbon Spheres for Efficient Chromium Ion Adsorption in Acidic Wastewater
Source: Nanomaterials (Basel). 2026 May 26;16(11):669. doi: 10.3390/nano16110669 (PMC13257643; doi:10.3390/nano16110669)
Supplement: Supplementary file 1 [file nanomaterials-16-00669-s001.zip › nanomaterials-4325494-supplementary.pdf]

Article

High-Specific-Surface-Area Hollow Carbon Spheres for Efficient Chromium Ion Adsorption in Acidic Wastewater

Rui Gao [1], Man Zhang [1], Xiaoyu Sun [1], Dongyang Zhu [1], Xin Huang [1], Ting Wang [1], Chuang Xie [1], Na Wang [1,*] and Hongxun Hao [1,2,3,*]

[1] National Engineering Research Center of Industry Crystallization Technology, School of Chemical Engineering and Technology, Tianjin University, Tianjin 300072, China
[2] State Key Laboratory of Chemical Engineering and Low-Carbon Technology, Shanghai 200237, China
[3] Engineering Research Center of Green Purification Process, Ministry of Education, Tianjin University, Tianjin 300072, China
* Correspondence: wangna224@tju.edu.cn (N.W.); hongxunhao@tju.edu.cn (H.H.)

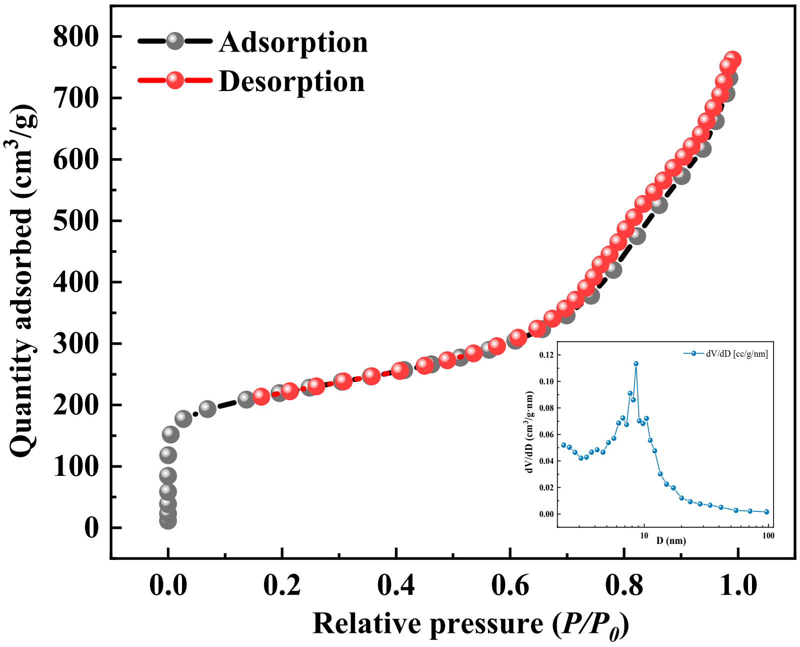

Figure S1 BET curve of HCM

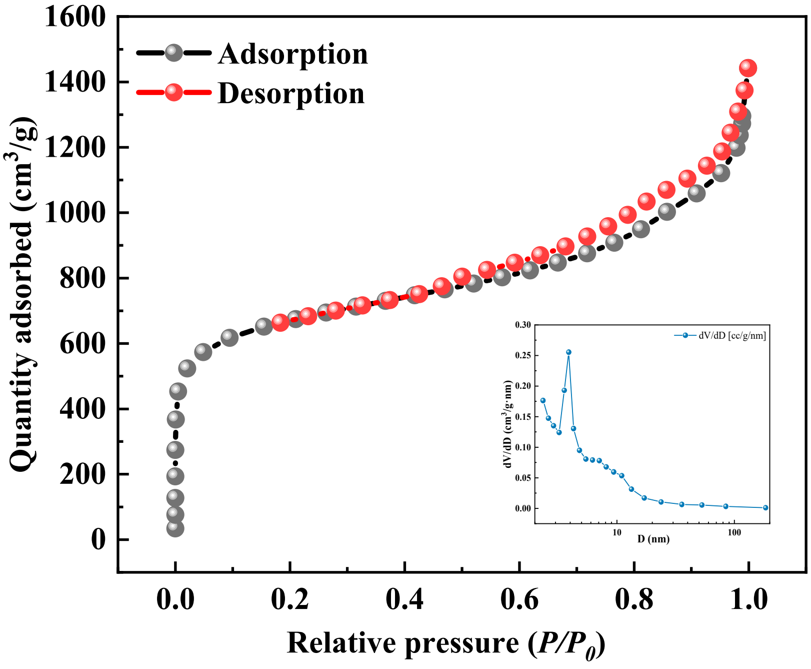

Figure S2 BET curve of $HCM_{2.5}$

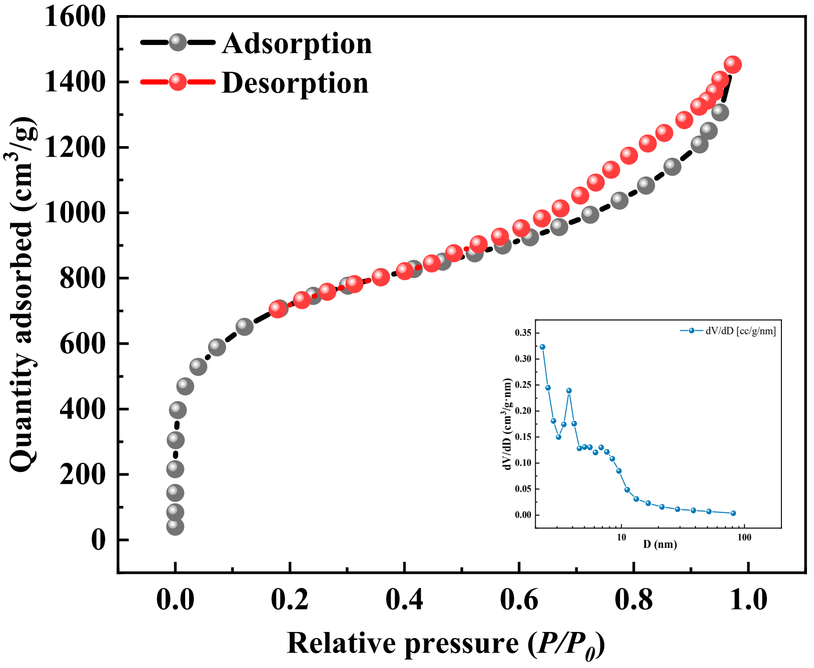

Figure S3 BET curve of HCM_5

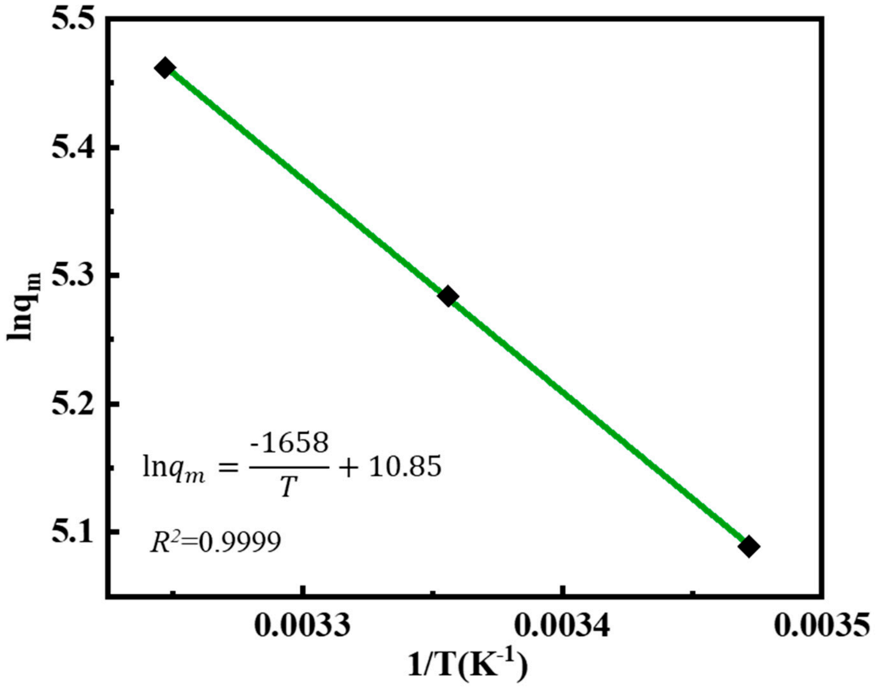

Figure S4 The thermodynamic fitting curve to calculate *ΔS* and *ΔH*

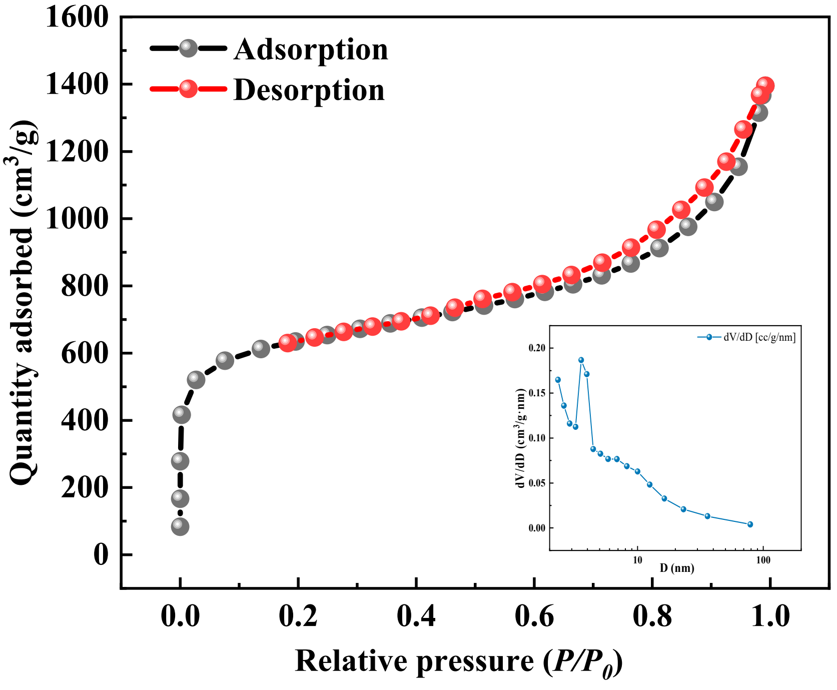

Figure S5 BET curve of $HCM_{2.5}$(Cycle number=1)

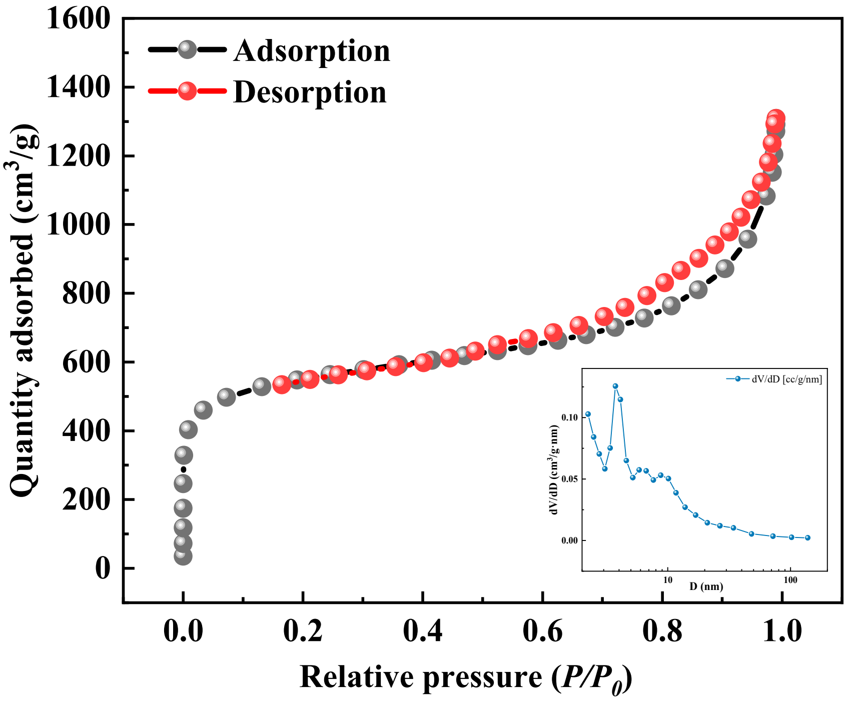

Figure S6 BET curve of $HCM_{2.5}$(Cycle number=2)
